# Fundamental absorption bandwidth to thickness limit for transparent homogeneous layers

**DOI:** 10.1515/nanoph-2023-0920

**Published:** 2024-03-08

**Authors:** Willie J. Padilla, Yang Deng, Omar Khatib, Vahid Tarokh

**Affiliations:** Pratt School of Engineering, Duke University, Durham, NC, 27705, USA

**Keywords:** fundamental limit, absorber, bandwidth

## Abstract

Past work has considered the analytic properties of the reflection coefficient for a metal-backed slab. The primary result established a fundamental relationship for the minimal layer thickness to bandwidth ratio achievable for an absorber. There has yet to be establishment of a similar relationship for non-metal-backed layers, and here we present the universal result based on the Kramers–Kronig relations. Our theory is validated with transfer matrix calculations of homogeneous materials, and full-wave numerical simulations of electromagnetic metamaterials. Our results place more general fundamental limits on absorbers and thus will be important for both fundamental and applied studies.

## 1 Introduction

The quantities of absorption and emission are fundamental processes related to the energy contribution of radiative heat transfer. Kirchhoff’s law of thermal radiation derives from the second law of thermodynamics and indicates that the emissivity (E) and absorptivity (A) of a surface are identical in thermal equilibrium over spectral, directional, and solid angle degrees of freedom [1]. Therefore, for any system operating in thermal equilibrium an understanding of the fundamental limits placed on absorption is of paramount importance. Physical bounds offer insights into the peak performance an absorber can achieve, given a specific material type and size. The potential of universally derived bounds, irrespective of specific materials, can be significant in developing and analyzing various absorber types. These bounds can further serve as benchmarks for evaluating the performance and stopping criteria in optimization of absorber bandwidth [2], [3], [4], [5], [6]. Only after gaining a thorough understanding of the fundamental limits of absorption can more complex scenarios in radiative transfer be explored.

One fundamental limit established the relationship between absorber thickness and absorption bandwidth, and is valid for any single-layer (or multi-layer) metal backed structure. An assumption is that the reflection coefficient can be described in the long wavelength limit, keeping only the first-order term in *λ* ^−1^, which is given as,

(1) $$\tilde{r}\left(\lambda \right){|}_{\lambda \to \infty }=-1+i\frac{4\pi \mu d}{\lambda_{0}}$$

Using the reflection coefficient given in Eq. (1), a Kramers–Kronig analysis shows that the limit for a metal-backed absorber of thickness (*d*_*RL*_) is given by [7],

(2) $$d_{RL}\geq \frac{1}{2\pi^{2}\mu_{s}}\left|\overset{\infty }{\underset{0}{\int }}\ln|\tilde{r}\left(\lambda \right)|\;d\lambda \right|\equiv d_{R}$$

where *μ*_*s*_ = Re{*μ*}|_*λ*→∞_ is the static permeability of the absorber. Other works have followed the approach presented in [7] and have shown similar bounds [8], [9] for: materials with open boundary conditions [10], high impedance surfaces [8], antenna arrays [11], [12], [13], and metamaterials [14], [15], [16].

Here we derive a fundamental relationship between the absorption bandwidth and thickness for general transparent homogeneous layers. We consider the analytic properties of both the reflection and transmission coefficients which results in a dispersion relationship connecting the slab thickness and the static permeability and permittivity.

## 2 Transfer matrix for a single layer

Consider a homogeneous slab of matter of thickness *d* embedded in vacuum with material parameters *ϵ* = *ϵ*_*r*_ *ϵ*_0_ and *μ* = *μ*_*r*_ *μ*_0_, where *ϵ*_*r*_ and *μ*_*r*_ are the relative quantities, and *ϵ*_0_ and *μ*_0_ are the vacuum values. The reflection coefficient (*r*) and transmission coefficient (*t*) are determined using the transfer matrix method and at normal incidence they are,

(3) $$r=\frac{-\frac{i}{2}\left[Z_{r}^{-1}-Z_{r}\right]\sin\left(nk_{0}d\right)}{\cos\left(nk_{0}d\right)+\frac{i}{2}\left[Z_{r}^{-1}+Z_{r}\right]\sin\left(nk_{0}d\right)}$$

and

(4) $$t=\frac{1}{\cos\left(nk_{0}d\right)+\frac{i}{2}\left[Z_{r}^{-1}+Z_{r}\right]\sin\left(nk_{0}d\right)}$$

where $n=\sqrt{\mu_{r}\epsilon_{r}}$ is the refractive index, $Z_{r}=\sqrt{\mu_{r}/\epsilon_{r}}$ is the relative impedance, and *k*_0_ = 2*π*/*λ*_0_ is the free-space wavevector where *λ*_0_ is the free-space wavelength.

Notice that *r* and *t* are related, i.e. they can be written [17],

(5) $$\frac{r}{t}=-\frac{i}{2}\left[Z_{r}^{-1}-Z_{r}\right]\sin\left(nk_{0}d\right)$$

The magnitude squared of Eq. (5) is therefore,

(6) $${\left|\frac{r}{t}\right|}^{2}=\frac{1}{4}\left[{\left|Z_{r}^{-1}-Z_{r}\right|}^{2}\right]{\left|\sin\left(nk_{0}d\right)\right|}^{2}$$

Equation (6) is the main result from the transfer matrix that we use for *r* and *t* to bound the absorption.

## 3 High frequency limit of material parameters

We specify the values of material parameters – where they exist – at infinite frequency (*ω* = ∞), or zero wavelength. All material parameters at zero wavelength, or infinite frequency, must take on the values of free-space in the classical limit. Specifically,

(7) $$\epsilon_{r}\left(\omega \right){|}_{\omega \to \infty }=1\;\text{or}\;\epsilon_{r}\left(\lambda \right){|}_{\lambda \to 0}=1$$

(8) $$\mu_{r}\left(\omega \right){|}_{\omega \to \infty }=1\;\text{or}\;\mu_{r}\left(\lambda \right){|}_{\lambda \to 0}=1$$

(9) $$n\left(\omega \right){|}_{\omega \to \infty }=1\;\text{or}\;n\left(\lambda \right){|}_{\lambda \to 0}=1$$

(10) $$Z_{r}\left(\omega \right){|}_{\omega \to \infty }=1\;\text{or}\;Z_{r}\left(\lambda \right){|}_{\lambda \to 0}=1$$

where the imaginary portion of each of these parameters is zero.

## 4 Details of calculations and the dispersion relation

Calculations in Ref. [7] are mainly focused on bounding $\int_{0}^{\infty }\ln\left(\left|r\left(\lambda \right)\right|\right)d\lambda$ since in that case |*r*(*λ*)|^2^ = 1 − *A*(*λ*), where *A*(*λ*) is the absorption in wavelength *λ*. In our case 1 − *A*(*λ*) = |*r*(*λ*)|^2^ + |*t*(*λ*)|^2^, where *t*(*λ*) is the transmission coefficient at wavelength *λ*. In this light, we focus on bounding $\int_{0}^{\infty }\ln\left(|r\left(\lambda \right){|}^{2}+|t\left(\lambda \right){|}^{2}\right)d\lambda$ instead. Clearly

$$\ln\left(|r\left(\lambda \right){|}^{2}+|t\left(\lambda \right){|}^{2}\right)=\ln\left(|t\left(\lambda \right){|}^{2}\right)+\ln\left[1+{\left|\frac{r\left(\lambda \right)}{t\left(\lambda \right)}\right|}^{2}\right].$$

Thus invoking Eq. (6) we find,

$$\begin{array}{ll} & \overset{\infty }{\underset{0}{\int }}\ln\left(|r\left(\lambda \right){|}^{2}+|t\left(\lambda \right){|}^{2}\right)d\lambda \\ & \;=\overset{\infty }{\underset{0}{\int }}\ln\left(|t\left(\lambda \right){|}^{2}\right)d\lambda +\overset{\infty }{\underset{0}{\int }}\ln\left[1+\frac{1}{4}\left[|Z_{r}^{-1}-Z_{r}{|}^{2}\right]\right.\\ & \;\times \left.|\sin\left(nk_{0}d\right){|}^{2}\right]d\lambda \end{array}$$

We note that |*r*(*λ*)|^2^ + |*t*(*λ*)|^2^ ≤ 1 thus $\int_{0}^{\infty }\ln\left(|t\left(\lambda \right){|}^{2}\right)d\lambda \leq 0$ and $\int_{0}^{\infty }\ln\left(|r\left(\lambda \right){|}^{2}+|t\left(\lambda \right){|}^{2}\right)d\lambda \leq 0$ . Additionally,

$$\overset{\infty }{\underset{0}{\int }}\ln\left(|t\left(\lambda \right){|}^{2}\right)d\lambda \leq \overset{\infty }{\underset{0}{\int }}\ln\left(|r\left(\lambda \right){|}^{2}+|t\left(\lambda \right){|}^{2}\right)d\lambda \leq 0$$

assuming that (as we will prove later) these integrals exist.

We next focus on $\int_{0}^{\infty }\ln\left(|t\left(\lambda \right){|}^{2}\right)d\lambda$ . The function *t*(*λ*) is causal and therefore analytic in the upper half-plane [18]. This analyticity implies that any poles present in the upper half-plane of ln(*t*(*λ*)) arise solely from the zeros of *t*(*λ*). As *λ* approaches 0, the slab’s impedance approaches a value of *Z*_*r*_ = *Z*_0_ = 1, leading *t* to assume the form e^*i*∞^. This infinite phase behavior at *t*(*λ* = 0) introduces an intrinsic singularity for ln(*t*(*λ*)), which is distinct from other poles associated with zeros of *t*(*λ*).

**Figure 1:**
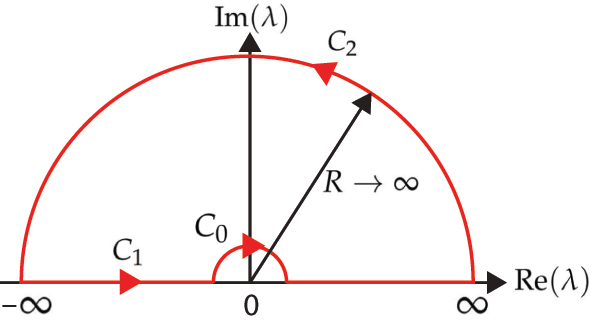
Contour used for integration of Eq. (11).

Therefore, to evaluate $\int_{0}^{\infty }\ln\left(|t\left(\lambda \right){|}^{2}\right)d\lambda$ we perform a contour integral over the composite path *C* = *C*_0_ + *C*_1_ + *C*_2_ as illustrated in Figure 1. As shown in the Supplementary Information Section 6, the contribution due to the intrinsic singularity (*C*_0_ term) gives a residue of $2\pi^{2}d\sqrt{\epsilon_{\infty }\mu_{\infty }}$ , where *ϵ*_∞_ ≡ *ϵ*_*λ*→0_, *μ*_∞_ ≡ *μ*_*λ*→0_, and both *ϵ*_∞_ and *μ*_∞_ are real.

To address poles of ln(*t*(*λ*)) which arise from the zeros of *t*(*λ*), we assume *n* poles in the upper half-plane, denoted as *λ*_1_, *λ*_2_, *…*, *λ*_*n*_. Using a product expansion [19] we multiply *t*(*λ*) with a Blaschke product and define an ancillary function as,

$$t^{\prime }\left(\lambda \right)\equiv t\left(\lambda \right)\overset{n}{\underset{i}{\prod }}\frac{\left(\lambda -\lambda_{i}^{*}\right)}{\left(\lambda -\lambda_{i}\right)}\equiv t\left(\lambda \right)B\left(\lambda \right)$$

that has neither poles nor zeros in the upper half plane, where λ* denotes the complex conjugate of *λ*. ln(*t*′(*λ*)) is analytic in the upper half plane of *λ* since *t*′(*λ*) does not have zeros in the upper half plane. Thus, from Cauchy’s integral theorem, we have

(11) $$\underset{C}{\oint }\ln\left(t^{\prime }\left(\lambda \right)\right)\;d\lambda =0$$

Taking the real part of the integral along paths *C* = *C*_0_ + *C*_1_ + *C*_2_, and noting that $R\left\{\ln\left[t^{\prime }\left(\lambda \right)\right]\right\}=|\ln\left[t^{\prime }\left(\lambda \right)\right]|=|\ln\left[t\left(\lambda \right)\right]|$ with the evenness of |ln[*t*(*λ*)]|, we have

$$\begin{array}{ll} & 2\overset{\infty }{\underset{0}{\int }}|\ln\left(t\left(\lambda \right)\right)|d\lambda +R\underset{C_{0}}{\oint }\ln\left(t\left(\lambda \right)\right)d\lambda +R\underset{C_{2}}{\oint }\ln\left(t\left(\lambda \right)\right)d\lambda \\ & \;+R\underset{C_{0}}{\oint }\ln\left(B\left(\lambda \right)\right)d\lambda +R\underset{C_{2}}{\oint }\ln\left(B\left(\lambda \right)\right)d\lambda =0 \end{array}$$

Building on the prior discussion and to simplify our analysis further, we will make the following assumptions: the limits of lim_*λ*→∞_ *ϵ*_*r*_(*λ*) ≡ *ϵ*_*s*_ and lim_*λ*→∞_ *μ*_*r*_(*λ*) ≡ *μ*_*s*_ exist.

Under the above assumption, as |*λ*| → ∞, we may approximate Eq. (4) as,

(12) $$t\left(\lambda \right)∼1-i\frac{\pi d\left(\epsilon_{s}+\mu_{s}\right)}{\lambda }+O\left(\frac{1}{\lambda^{2}}\right)$$

where $O\left(\frac{1}{\lambda^{2}}\right)$ denotes terms of order $\frac{1}{\lambda^{k}}$ for *k* ≥ 2 in the Laurent expansion of *t*(*λ*) around the point at infinity in *P* ^+^.

We next calculate $R\left(\oint_{C_{2}}\ln\left(t\left(\lambda \right)\right)d\lambda \right)$ as *R* → ∞. Letting *λ* = *R*exp(*iθ*) where *θ* varies from 0 to *π*, we have *dλ* = *iλdθ*. As *R* grows large, using Eq. (12) we have

$$\ln\left(t\left(\lambda \right)\right)∼\ln\left[1-i\frac{\pi d\left(\epsilon_{s}+\mu_{s}\right)}{\lambda }+O\left(\frac{1}{\lambda^{2}}\right)\right].$$

Simple manipulation gives

$$\underset{R\to \infty }{\lim}R\underset{C_{2}}{\oint }\ln\left(t\left(\lambda \right)\right)d\lambda =\pi^{2}dR\left(\epsilon_{s}+\mu_{s}\right)$$

We use a similar approach to evaluate $R\oint_{C_{0}}\ln\left[t^{\prime }\left(\lambda \right)\right]d\lambda$ and $R\oint_{C_{2}}\ln\left[t^{\prime }\left(\lambda \right)\right]d\lambda$ as *R* → 0 and *R* → ∞, respectively. Simple manipulation gives

$$\begin{array}{ll} \underset{R\to 0}{\lim}\;R\underset{C_{0}}{\oint }\ln\left[B\left(\lambda \right)\right]d\lambda & =0\\ \underset{R\to \infty }{\lim}\;R\underset{C_{2}}{\oint }\ln\left[B\left(\lambda \right)\right]d\lambda & =-2\pi \underset{i}{\sum }I\left(\lambda_{i}\right) \end{array}$$

Plugging the contribution of each term back into Eq. (11), we have

$$\begin{array}{ll} \overset{\infty }{\underset{0}{\int }}\ln\left(|t\left(\lambda \right){|}^{2}\right)d\lambda & =-\pi^{2}\left(\epsilon_{s,r}+\mu_{s,r}\right)d+2\pi^{2}d\sqrt{\epsilon_{\infty }\mu_{\infty }}\\ & \;+2\pi \underset{i}{\sum }I\left(\lambda_{i}\right) \end{array}$$

where $\epsilon_{s,r}\equiv R\left(\epsilon_{s}\right)$ and $\mu_{s,r}\equiv R\left(\mu_{s}\right)$ . Since $I\left(\lambda_{i}\right)>0$ in the upper half plane of *λ*

$$\overset{\infty }{\underset{0}{\int }}\ln\left(|t\left(\lambda \right)\right){|}^{2})d\lambda \geq -\pi^{2}\left(\epsilon_{s,r}+\mu_{s,r}\right)d+2\pi^{2}d\sqrt{\epsilon_{\infty }\mu_{\infty }}$$

It follows that

$$\begin{array}{ll} -\pi^{2}\left[\epsilon_{s,r}+\mu_{s,r}-2\sqrt{\epsilon_{\infty }\mu_{\infty }}\right]d & \leq \overset{\infty }{\underset{0}{\int }}\ln\left(|t\left(\lambda \right){|}^{2}\right)d\lambda \\ & \leq \overset{\infty }{\underset{0}{\int }}\ln\left(|r\left(\lambda \right){|}^{2}+|t\left(\lambda \right){|}^{2}\right)d\lambda \\ & \leq 0 \end{array}$$

which gives

$$\left|\overset{\infty }{\underset{0}{\int }}\ln\left(|r\left(\lambda \right){|}^{2}+|t\left(\lambda \right){|}^{2}\right)d\lambda \right|\leq \pi^{2}\left|\epsilon_{s,r}+\mu_{s,r}-2\sqrt{\epsilon_{\infty }\mu_{\infty }}\right|d.$$

or in terms of the material thickness – redefined as *d*_*TL*_,

(13) $$d_{TL}\geq \frac{\left|\overset{\infty }{\underset{0}{\int }}\ln\left[|r\left(\lambda \right){|}^{2}+|t\left(\lambda \right){|}^{2}\right]d\lambda \right|}{\pi^{2}\left|\epsilon_{s,r}+\mu_{s,r}-2\sqrt{\epsilon_{\infty }\mu_{\infty }}\right|}\equiv d_{T}$$

This is an analog of the bound shown in [7] and can be used in a corresponding manner to bound the absorption bandwidth as a function of thickness of transparent homogeneous materials.

In particular if the absorption *A*(*λ*) of the material between wavelengths [*λ*_0_, *λ*_1_] satisfies *A*(*λ*) > 1 − *δ* for some 0 < *δ* < 1, then |*r*(*λ*)|^2^ + |*t*(*λ*)|^2^ ≤ *δ* in [*λ*_0_, *λ*_1_] and it follows that

$$\begin{array}{ll} |\ln\left(\delta \right)|\left(\lambda_{1}-\lambda_{0}\right) & \leq \left|\overset{\infty }{\underset{0}{\int }}\ln\left(|r\left(\lambda \right){|}^{2}+|t\left(\lambda \right){|}^{2}\right)d\lambda \right|\\ & \leq \pi^{2}|\left(\epsilon_{s,r}+\mu_{s,r}\right)-2\sqrt{\epsilon_{\infty }\mu_{\infty }}|d \end{array}$$

and the thickness to absorption bandwidth is given by,

(14) $$d\geq \frac{\lambda_{1}-\lambda_{0}}{\pi^{2}}\left|\frac{\ln\delta }{\epsilon_{s,r}+\mu_{s,r}-2\sqrt{\epsilon_{\infty }\mu_{\infty }}}\right|$$

## 5 Validation

We next validate Eq. (13) for two test cases – a homogeneous material of thickness *d* modeled as a Lorentz oscillator, and numerical simulations of metamaterials.

### 5.1 Lorentz oscillator model

**Figure 2:**
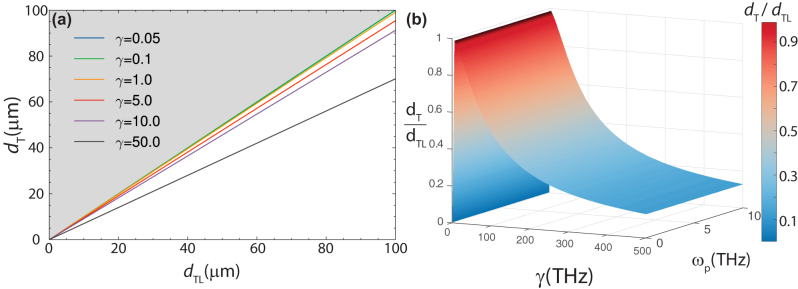
Calculated values of *d*_*T*_ using a Lorentz model for a slab of magneto-dielectric material of thickness *d*_*TL*_. In (a) the curves are for different damping frequencies *γ*, as described in the text. In (b), we plot the ratio *d*_*T*_/*d*_*TL*_ as a function of *γ* and *ω*_*p*_ for the special case when *ϵ*(*ω*) = *μ*(*ω*).

We consider a general magneto-dielectric slab where the frequency dependent relative permittivity (*ϵ*(*ω*)) and permeability (*μ*(*ω*)) material parameters are represented by a Lorentz oscillator model:

$$\begin{array}{ll} \epsilon \left(\omega \right) & =\epsilon_{\infty }+\frac{\omega_{p}^{2}}{\omega_{0}^{2}-\omega^{2}-i\gamma \omega }\\ \mu \left(\omega \right) & =\mu_{\infty }+\frac{\omega_{p,m}^{2}}{\omega_{0,m}^{2}-\omega^{2}-i\gamma_{m}\omega } \end{array}$$

where *ϵ*_∞_, *μ*_∞_ are the permittivity and permeability, respectively, at infinite frequency, *ω* is the angular frequency, *ω*_*p*_, *ω*_*p*,*m*_ are the plasma frequencies, *ω*_0_, *ω*_0,*m*_ are the resonant frequencies, and *γ*, *γ*_*m*_ are the damping frequencies. In Figures 2 [Fig j_nanoph-2023-0920_fig_003]–4, Lorentz parameters have units of 2*π* × THz. We use a sub-scripted “*m*” to distinguish the Lorentz parameters for the *μ* oscillator.

**Figure 3: j_nanoph-2023-0920_fig_003:**
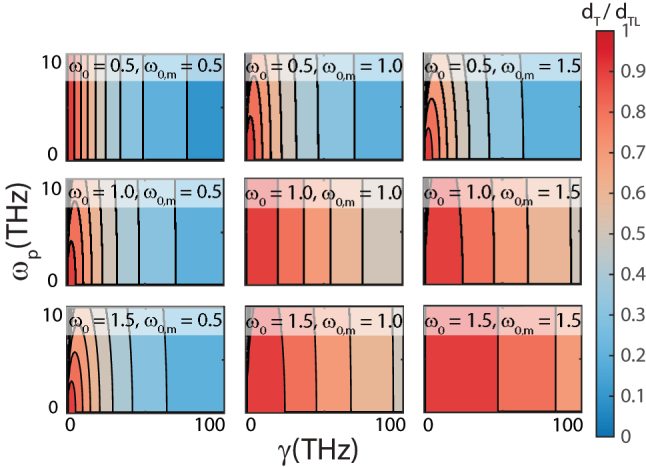
Contour plots of *d*_*T*_/*d*_*TL*_ as a function of *ω*_*p*_ and *γ*, from the Lorentz model where *ω*_*p*_ = *ω*_*p*,*m*_ and *γ* = *γ*_*m*_. Each plot corresponds to a different pair of *ω*_0_ and *ω*_0,*m*_, showcasing the dependence of the ratio on the plasma frequency and damping frequency under a general Lorentz model.

**Figure 4:**
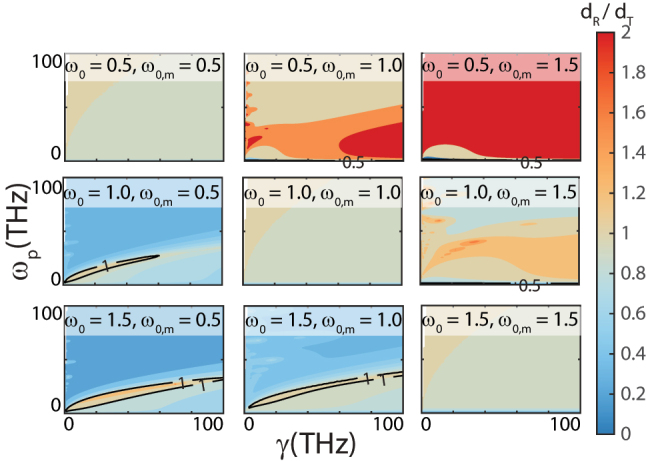
Contour plots of *d*_*R*_/*d*_*T*_ as a function of *ω*_*p*_ and *γ*, from the Lorentz model where *ω*_*p*_ = *ω*_*p*,*m*_ and *γ* = *γ*_*m*_. Each plot corresponds to a different pair of *ω*_0_ and *ω*_0,*m*_, showcasing the dependence of the ratio on the plasma frequency and damping frequency under a general Lorentz model.

We first assign equal permittivity and permeability values *ϵ*(*ω*) = *μ*(*ω*), such that the slab impedance matches free space, i.e. *Z*_*r*_ = 1. Therefore from Eqs. (3) and (4) we find,

(15) r(λ)=0

(16) $$t\left(\lambda \right)=\frac{1}{\cos\left(nk_{0}d\right)+i\sin\left(nk_{0}d\right)}$$

The slab thickness in the TMM is given as *d* = *d*_*TL*_ in Eq. (13). Our objective is to verify the fundamental bound for transparent layers which asserts that *d*_*TL*_ ≥ *d*_*T*_. Here *d*_*T*_ is calculated using the right side of Eq. (13), with *r*(*λ*) and *t*(*λ*) derived from Eqs. (15) and (16), under the assumption that *ϵ*_*s*_ = *ϵ*(*ω* = 0) and *μ*_*s*_ = *μ*(*ω* = 0). The slab is given as: *ϵ*_∞_ = 1.0, *ω*_0_ = 2*π* × 1.0 THz, *ω*_*p*_ = 2*π* × 1.25 THz, and *γ* = 2*π* × 0.05 THz. In Figure 2(a), we explore a range of damping frequencies (solid curves) from *γ* = 2*π* × 0.05 THz to *γ* = 2*π* × 50 THz, plotting *d*_*TL*_ against *d*_*T*_ while holding other parameters constant. None of the curves lie in the shaded gray area (where *d*_*T*_ > *d*_*TL*_), which confirms the soundness of the specified limit.

To further validate Eq. (13), we examined the ratio *d*_*T*_/*d*_*TL*_ across a broad range of values for *γ* and *ω*_*p*_, as depicted in Figure 2(b). Notably, for an impedance-matched slab, the real part of the residue solely depends on *γ* and is unaffected by *ω*_*p*_. Across all the examined parameters, the value of *d*_*T*_/*d*_*TL*_ consistently remained below one, reinforcing the fundamental limit. Moreover, Figure 2 showcases an inverse correlation between *d*_*T*_ and *γ*. This observation suggests that a transmissive slab, when impedance-matched in free space, adheres to the anticipated absorptance behavior. As *γ* decreases, the residual of ln(*t*) also diminishes, aligning the slab closer to the fundamental bound established by Eq. (13).

While an impedance-matched transmissive slab provides substantial validation for Eq. (13), it’s important to note that our initial testing focused solely on the *ϵ*(*ω*) = *μ*(*ω*) case. To broaden the scope of our verification process, we extended our test cases to include transmissive slabs with variable and non-equal *ϵ*(*ω*) and *μ*(*ω*) values. Figure 3 showcases contour plots of the *d*_*T*_/*d*_*TL*_ ratio as a function of *γ* and *ω*_*p*_, where *ω*_*p*_ = *ω*_*p*,*m*_ and *γ* = *γ*_*m*_. Each of these plots represent a unique combination of resonance frequencies for *ϵ*(*ω*) and *μ*(*ω*). It becomes evident that altering the resonance frequency results in noticeable variations in *d*_*T*_ across the domain. However, the bound as defined in Eq. (13), specifically *d*_*TL*_ ≥ *d*_*T*_, remains unviolated.

Our research also investigates the relationship between the two fundamental limits discussed here, i.e. that of a transparent slab compared to that of a metal backed slab. The latter is defined on the right side of Eq. (2) and denoted as *d*_*R*_. Figure 4 displays contour plots of the ratio *d*_*R*_/*d*_*T*_ as a function of *γ* and *ω*_*p*_, where *ω*_*p*_ = *ω*_*p*,*m*_ and *γ* = *γ*_*m*_. Each contour plot accounts for a distinct combination of *ω*_0_ and *ω*_0,*m*_. The comparison of the differences between *d*_*R*_ and *d*_*T*_ is intriguing as we set the thickness to be identical for both metal-backed and transmissive slabs, i.e. *d*_*TL*_ = *d*_*RL*_.

When both slabs are impedance-matched to free space, i.e. *ω*_0_ = *ω*_0,*m*_, Figure 4 shows that *d*_*R*_ ≅ *d*_*T*_. This result may appear counterintuitive at first glance. Namely, when the two cases share identical material properties, the difference in absorption is solely governed by the optical path length within the slab. Considering that the metal-backed slab reflects the wave, the optical path length is double that of the transmissive slab. Yet, our findings elucidate this seemingly unconventional behavior. Our derivation of the fundamental limit diverges from the metal-backed limit approach by emphasizing reflectance *R* = |*r*|^2^ and transmittance *T* = |*t*|^2^ rather than just the reflection coefficient *r*. In this context, the natural logarithm of the reflection coefficient is half the natural log of the reflectance. This aspect effectively counterbalances the doubled optical path length inherent in a metal-backed slab, resulting in a *d*_*R*_/*d*_*T*_ ratio that approaches unity. However, when *ω*_0_ ≠ *ω*_0,*m*_, *d*_*R*_/*d*_*T*_ deviates from unity. For *ω*_0_ < *ω*_0,*m*_, *d*_*R*_/*d*_*T*_ may exceed 1 indicating a metal-backed slab achieves greater absorption bandwidth, while for *ω*_0_ > *ω*_0,*m*_ *d*_*R*_/*d*_*T*_ largely remains below 1 where the transparent slab yields more absorption bandwidth. By elucidating these concepts, we aim to deepen the understanding of the relationship between Eqs. (13) and (2), as well as the behaviors observed in Figure 4.

### 5.2 Electromagnetic metamaterials

Subwavelength metallic metamaterials play a pivotal role in absorbing electromagnetic radiation for myriad applications [20], [21]. Traditional configurations use a metallic split-ring resonator near a continuous ground plane [22], [23], which enhances performance by focusing light within a limited volume. Recent studies indicate that resonant modes supported by all-dielectric resonators also achieve subwavelength localization and high absorption [24], [25]. By exploiting the frequency degeneracy of these modes through careful metasurface design, highly efficient absorbers can be created. The use of metallic and dielectric materials exemplifies two fundamental strategies for constructing metasurface absorbers, and the absorption bandwidth serves as a critical metric for both systems. The limit established for a metal-back slab defines a core link between absorber thickness and absorption bandwidth [7]. However, no universal theory currently exists outlining the relationship between absorption bandwidth and the absorber thickness for non-metal-backed materials despite numerous theoretical, computational, and experimental studies [20], [26]. Our results presented in Eq. (13) introduces a fundamental thickness limit to the absorption bandwidth for metasurface absorbers, paving the way for more efficient design of transmissive absorbers.

**Figure 5:**
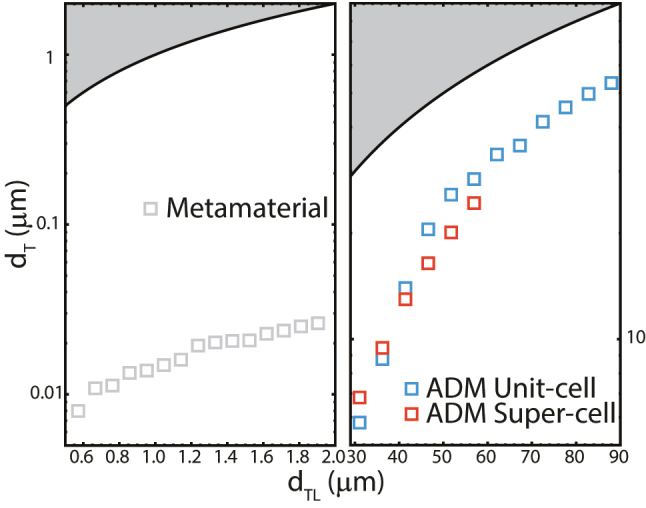
Calculated *d*_*T*_ from metamaterial absorbers of thickness *d*_*TL*_. The ADM unit cell is blue, the 2 × 2 ADM supercell is red. The metal-based unit-cell is shown as gray.

We focused our analysis on metal-based and all-dielectric metamaterial (ADM) absorbers using Eq. (13). For both types of representative metamaterial systems, we chose commonly used resonator geometries for the periodic unit cell, tuned to give near-unity peak absorptivity at 1 THz. We also considered a 2 × 2 supercell ADM, where two different unit cells can be combined to broaden the absorption bandwidth. We calculated *d*_*T*_ by averaging the effective material parameters at low frequencies using known S-parameter inversion techniques [27]. Details of the simulations and calculations can be found in the Supplementary Information Section 9. Our computations are presented in Figure 5 for both ADM (5 (a)) and metal-based (5 (b)) metamaterial absorbers. Interestingly, all the metamaterials explored fall well short of the fundamental thickness to bandwidth limit, a result of their narrow-band absorption. Notably, the 2 × 2 resonator supercell, expected to broaden the ADM’s absorptance peak, deviates further from the limit, suggesting higher values of *μ*_*s*_ and *ϵ*_*s*_.

## 6 Conclusions

Our investigation has extended fundamental relationships governing the absorption bandwidth and material thickness in non-metal backed layers. We have established a universal result based on the causal Kramers–Kronig relations, filling a significant gap in the current knowledge. This achievement was validated through transfer matrix calculations of homogeneous materials and full-wave numerical simulations of electromagnetic metallic and all-dielectric metamaterials, ensuring the robustness of our theory. Our results extend the known fundamental limits on absorbers, which is of crucial importance in both basic research and practical applications, and energy related fields stand to benefit greatly from this insight. We anticipate that this work will inform and inspire future studies in understanding the properties of absorptive materials and systems. We hope that our research can provide a robust theoretical foundation that can aid the design and development of more efficient energy harvesting and radiative systems, enabling more sustainable and cost-effective solutions to global energy needs.

## Supplementary Material

Supplementary Material Details

Supplementary Material Details

Supplementary Material Details

Supplementary Material Details
