# Supplementary figures and images for: Fundamental absorption bandwidth to thickness limit for transparent homogeneous layers
Source: Nanophotonics. 2024 Mar 8;13(9):1623–9. doi: 10.1515/nanoph-2023-0920 (PMC11636404; doi:10.1515/nanoph-2023-0920)

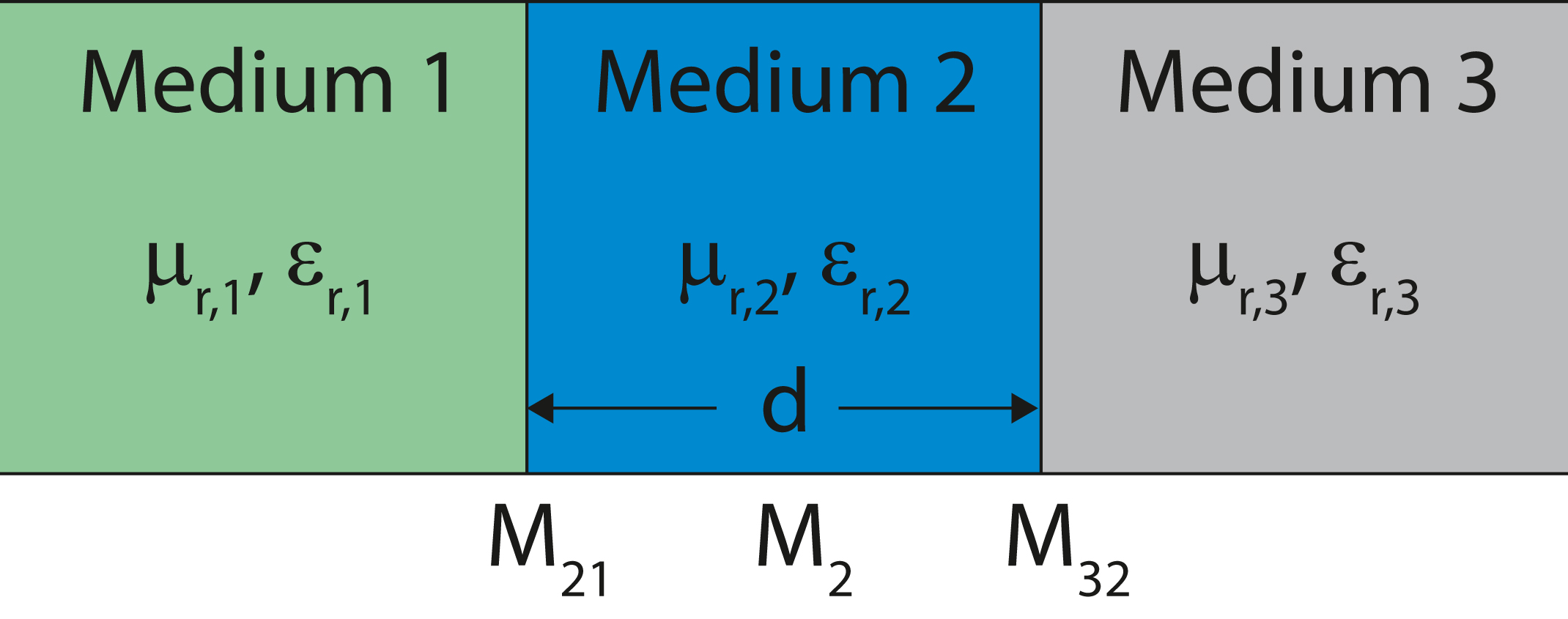

Supplement: Supplementary file 2 — Supplementary Material Details [file j_nanoph-2023-0920_suppl_002.jpg]

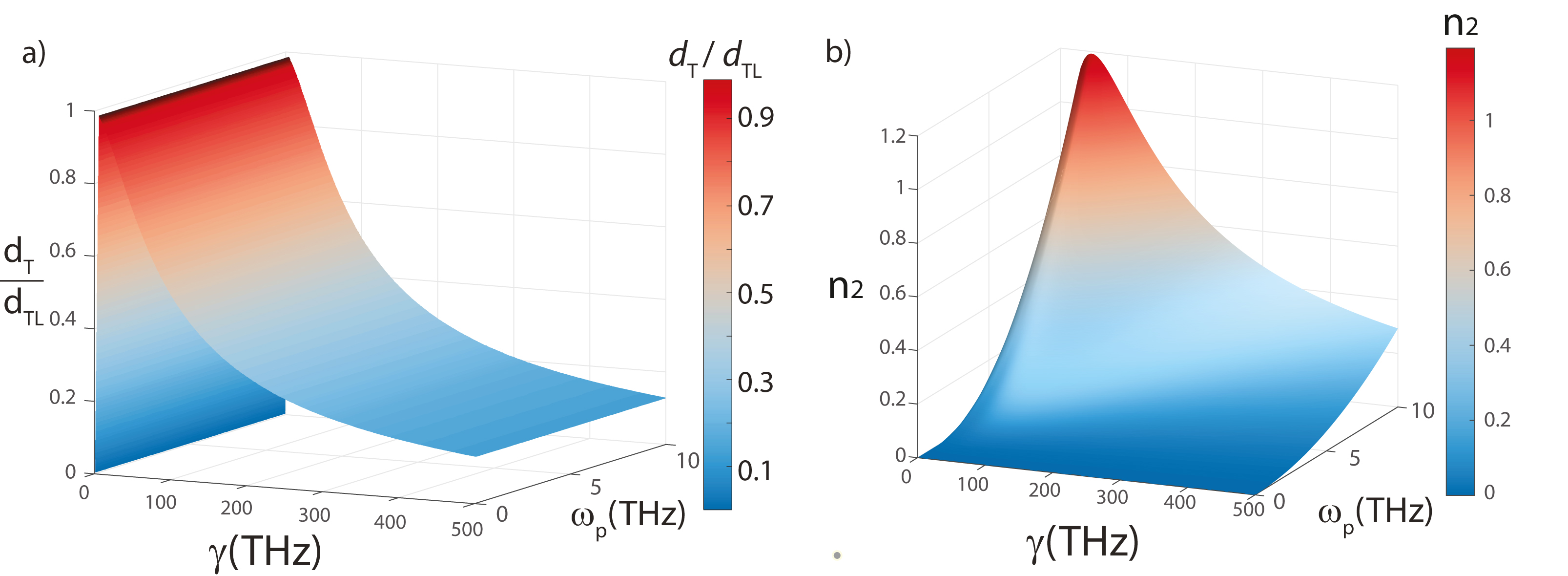

Supplement: Supplementary file 3 — Supplementary Material Details [file j_nanoph-2023-0920_suppl_003.jpg]

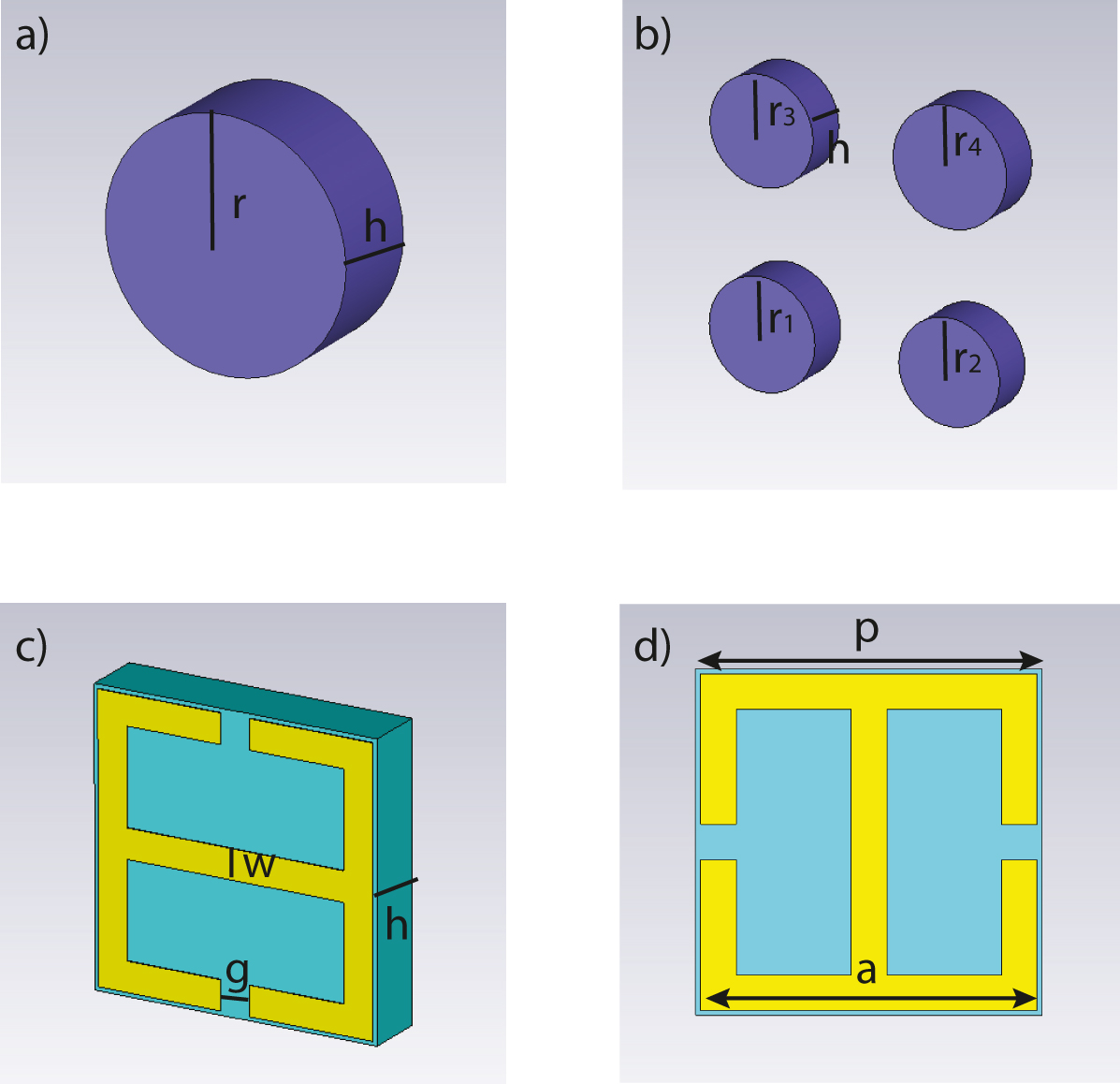

Supplement: Supplementary file 4 — Supplementary Material Details [file j_nanoph-2023-0920_suppl_004.jpg]
